# Dimethyl 6-acetyl-2-methyl-1,2-dihydroquinoline-2,4-dicarboxyl­ate

**DOI:** 10.1107/S1600536812003650

**Published:** 2012-02-04

**Authors:** Zeynep Gültekin, Michael Bolte, Tuncer Hökelek

**Affiliations:** a Department of Chemistry, Çankırı Karatekin University, TR-18100 Çankırı, Turkey; b Institut für Anorganische Chemie, J. W. Goethe-Universität Frankfurt, Max-von-Laue-Strasse 7, D-60438 Frankfurt/Main, Germany; c Department of Physics, Hacettepe University, 06800 Beytepe, Ankara, Turkey

## Abstract

In the title compound, C_16_H_17_NO_5_, the dihydro­pyridine ring adopts a sofa conformation. In the crystal, inter­molecular N—H⋯O hydrogen bonds link the mol­ecules into chains running along the *b* axis.

## Related literature

For the methods reported in the literature for the preparation of 1,2-dihydro­quinolines, see: Hu *et al.* (2011 ▶); Yadav *et al.* (2007 ▶, 2008 ▶); Waldmann *et al.* (2008 ▶). For the biological activity of dihydro­quinolines, see: Craig & Pearson (1971 ▶); Muren & Weissman (1971 ▶); Hamann *et al.* (1998 ▶); He *et al.* (2003 ▶); LaMontagne *et al.* (1989 ▶). For related structures, see: Gültekin *et al.* (2010 ▶); Gültekin *et al.* (2011*a* ▶,*b* ▶). For ring-puckering parameters, see: Cremer & Pople (1975 ▶).
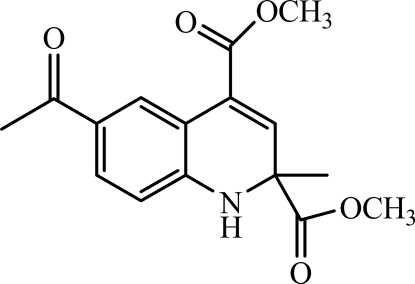


## Experimental

### 

#### Crystal data

C_16_H_17_NO_5_
*M* *_r_* = 303.31
Triclinic, 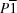

*a* = 7.9853 (7) Å
*b* = 8.3950 (7) Å
*c* = 12.4416 (11) Å
α = 89.308 (7)°
β = 74.436 (7)°
γ = 71.568 (7)°
*V* = 759.82 (12) Å^3^
*Z* = 2
Mo *K*α radiation
μ = 0.10 mm^−1^
*T* = 173 K
0.36 × 0.34 × 0.31 mm

#### Data collection

Stoe IPDS II two-circle diffractometer
12439 measured reflections
2833 independent reflections
2507 reflections with *I* > 2σ(*I*)
*R*_int_ = 0.044

#### Refinement

*R*[*F* ^2^ > 2σ(*F* ^2^)] = 0.049
*wR*(*F* ^2^) = 0.121
*S* = 1.05
2833 reflections
207 parameters
H atoms treated by a mixture of independent and constrained refinement
Δρ_max_ = 0.26 e Å^−3^
Δρ_min_ = −0.22 e Å^−3^

### 

Data collection: *X-AREA* (Stoe & Cie, 2001 ▶); cell refinement: *X-AREA*; data reduction: *X-AREA*; program(s) used to solve structure: *SHELXS97* (Sheldrick, 2008 ▶); program(s) used to refine structure: *SHELXL97* (Sheldrick, 2008 ▶); molecular graphics: *ORTEP-3 for Windows* (Farrugia, 1997 ▶); software used to prepare material for publication: *WinGX* (Farrugia, 1999 ▶) and *PLATON* (Spek, 2009 ▶).

## Supplementary Material

Crystal structure: contains datablock(s) I, global. DOI: 10.1107/S1600536812003650/xu5460sup1.cif

Structure factors: contains datablock(s) I. DOI: 10.1107/S1600536812003650/xu5460Isup2.hkl

Supplementary material file. DOI: 10.1107/S1600536812003650/xu5460Isup3.cml

Additional supplementary materials:  crystallographic information; 3D view; checkCIF report

## Figures and Tables

**Table 1:** Hydrogen-bond geometry (Å, °)

*D*—H⋯*A*	*D*—H	H⋯*A*	*D*⋯*A*	*D*—H⋯*A*
N1—H1⋯O5^i^	0.85 (2)	2.10 (2)	2.9223 (19)	166 (2)

Symmetry code: (i) 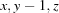.

**Fig. 1.**
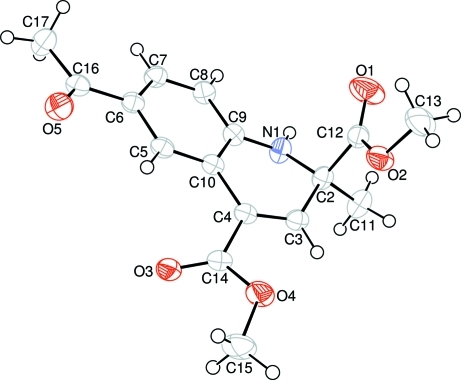
The molecular structure of the title compound with the atom-numbering scheme. Displacement ellipsoids are drawn at the 50% probability level.

**Fig. 2.**
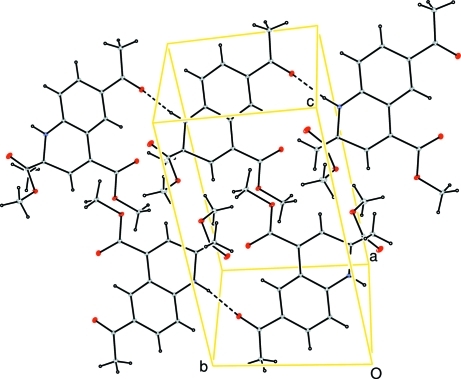
A partial packing diagram. Hydrogen bonds are shown as dashed lines.

## supplementary crystallographic information

### Comment

1,2-Dihydroquinoline derivatives have been considerably important for the preparation of biologically important compounds (Craig & Pearson, 1971; Muren & Weissman, 1971). Many methods have been reported in the literature for the preparation of 1,2-dihydroquinolines (Yadav *et al.*, 2007, 2008). The most convenient method is the condensation of aromatic amines with ketones using a catalytic amount of a Lewis acid or Brønsted acid (Hu *et al.*, 2011; Waldmann *et al.*, 2008). Dihydroquinolines are also powerful intermediates for the preparation of quinolines and many quinolines display biological effects (Hamann *et al.*, 1998; LaMontagne *et al.*, 1989; He *et al.*, 2003).

The structures of some 1,2-dihydroquinoline derivatives, C_16_H_19_NO_4_ (Gültekin *et al.*, 2010), C_14_H_15_NO_4_ (Gültekin *et al.*, 2011*a*) and C_17_H_21_NO_7_ (Gültekin *et al.*, 2011*b*) have also been determined.

In the title compound, (I), (Fig. 1), the ring A (C2–C4/C9/C10/N1) is not planar, but adopting a sofa conformation with puckering parameters (Cremer & Pople, 1975) Q_T_ = 0.348 (2)Å, φ = -45.4 (4)° and θ = 49.3 (3)°.

In the crystal structure, intermolecular N—H···O hydrogen bonds (Table 1) link the molecules to form infinite chains along the b-axis (Fig. 2).

### Experimental

The title compound was synthesized by the literature method (Waldmann *et al.*, 2008). p-acetyl aniline (100 mg, 1 eq) was dissolved in acetonitrile (1.5 ml), and then Bi(OTf)_3_ (5 mol%, 0.05 eq) and methyl pyruvate (2.2 eq) were added to the mixture. The mixture was heated by microwave irradiation for 3 h until the starting material was completely consumed as monitored by TLC. The resultant residue was directly purified by flash chromatography on silica (EtOAc:Cylohexane 1:2). Recrystallization over pentane and ethyl acetate (70:30) gave a yellow crystalline solid (yield: 91%), R_f_ 0.31 (2:1 Cyclohexane/EtOAc) m.p.: 425–426 K.

### Crystal data

**Table:**

C_16_H_17_NO_5_	*Z* = 2
*M**_r_* = 303.31	*F*(000) = 320
Triclinic, *P*1	*D*_x_ = 1.326 Mg m^−^^3^
Hall symbol: -P 1	Mo *K*α radiation, λ = 0.71073 Å
*a* = 7.9853 (7) Å	Cell parameters from 11792 reflections
*b* = 8.3950 (7) Å	θ = 3.5–25.9°
*c* = 12.4416 (11) Å	µ = 0.10 mm^−^^1^
α = 89.308 (7)°	*T* = 173 K
β = 74.436 (7)°	Block, light brown
γ = 71.568 (7)°	0.36 × 0.34 × 0.31 mm
*V* = 759.82 (12) Å^3^	

### Data collection

**Table:**

Stoe IPDS II two-circle diffractometer	2507 reflections with *I* > 2σ(*I*)
Radiation source: fine-focus sealed tube	*R*_int_ = 0.044
Graphite monochromator	θ_max_ = 25.6°, θ_min_ = 3.4°
ω scans	*h* = −9→9
12439 measured reflections	*k* = −10→10
2833 independent reflections	*l* = −15→15

### Refinement

**Table:**

Refinement on *F*^2^	Primary atom site location: structure-invariant direct methods
Least-squares matrix: full	Secondary atom site location: difference Fourier map
*R*[*F*^2^ > 2σ(*F*^2^)] = 0.049	Hydrogen site location: inferred from neighbouring sites
*wR*(*F*^2^) = 0.121	H atoms treated by a mixture of independent and constrained refinement
*S* = 1.05	*w* = 1/[σ^2^(*F*_o_^2^) + (0.0649*P*)^2^ + 0.2357*P*] where *P* = (*F*_o_^2^ + 2*F*_c_^2^)/3
2833 reflections	(Δ/σ)_max_ < 0.001
207 parameters	Δρ_max_ = 0.26 e Å^−^^3^
0 restraints	Δρ_min_ = −0.22 e Å^−^^3^

### Special details

**Table:**

Geometry. All esds (except the esd in the dihedral angle between two l.s. planes) are estimated using the full covariance matrix. The cell esds are taken into account individually in the estimation of esds in distances, angles and torsion angles; correlations between esds in cell parameters are only used when they are defined by crystal symmetry. An approximate (isotropic) treatment of cell esds is used for estimating esds involving l.s. planes.
Refinement. Refinement of F^2^ against ALL reflections. The weighted R-factor wR and goodness of fit S are based on F^2^, conventional R-factors R are based on F, with F set to zero for negative F^2^. The threshold expression of F^2^ > 2sigma(F^2^) is used only for calculating R-factors(gt) etc. and is not relevant to the choice of reflections for refinement. R-factors based on F^2^ are statistically about twice as large as those based on F, and R- factors based on ALL data will be even larger.

### Fractional atomic coordinates and isotropic or equivalent isotropic displacement parameters (Å^2^)

**Table:**

	*x*	*y*	*z*	*U*_iso_*/*U*_eq_	
O1	0.6972 (2)	−0.1203 (2)	0.18082 (12)	0.0633 (4)	
O2	0.69874 (15)	−0.00298 (16)	0.34026 (9)	0.0414 (3)	
O3	0.12719 (16)	0.61353 (14)	0.44347 (9)	0.0371 (3)	
O4	0.29430 (17)	0.43432 (16)	0.53946 (10)	0.0443 (3)	
O5	0.26090 (17)	0.80610 (14)	0.09807 (10)	0.0407 (3)	
N1	0.31769 (19)	0.06145 (17)	0.22877 (11)	0.0332 (3)	
H1	0.322 (3)	−0.019 (3)	0.1868 (16)	0.037 (5)*	
C2	0.4058 (2)	0.01893 (18)	0.31827 (12)	0.0297 (3)	
C3	0.34378 (19)	0.17245 (19)	0.39857 (12)	0.0286 (3)	
H3	0.3402	0.1582	0.4733	0.034*	
C4	0.29367 (18)	0.32849 (18)	0.36578 (12)	0.0271 (3)	
C5	0.27307 (19)	0.50723 (18)	0.20096 (12)	0.0278 (3)	
H5	0.2688	0.5999	0.2431	0.033*	
C6	0.26233 (19)	0.52543 (19)	0.09059 (12)	0.0285 (3)	
C7	0.2661 (2)	0.38508 (19)	0.02853 (12)	0.0312 (3)	
H7	0.2549	0.3960	−0.0439	0.037*	
C8	0.2861 (2)	0.23170 (19)	0.07329 (13)	0.0317 (3)	
H8	0.2896	0.1398	0.0307	0.038*	
C9	0.30114 (19)	0.21314 (18)	0.18284 (12)	0.0278 (3)	
C10	0.28990 (18)	0.35501 (18)	0.24904 (12)	0.0266 (3)	
C11	0.3554 (3)	−0.1283 (2)	0.37615 (15)	0.0416 (4)	
H11A	0.3912	−0.2219	0.3219	0.062*	
H11B	0.2252	−0.0943	0.4101	0.062*	
H11C	0.4185	−0.1609	0.4327	0.062*	
C12	0.6168 (2)	−0.04291 (18)	0.26972 (12)	0.0317 (3)	
C13	0.8977 (2)	−0.0641 (3)	0.30643 (17)	0.0581 (6)	
H13A	0.9424	−0.0192	0.3594	0.087*	
H13B	0.9422	−0.0287	0.2337	0.087*	
H13C	0.9403	−0.1849	0.3038	0.087*	
C14	0.2280 (2)	0.47500 (19)	0.45107 (12)	0.0291 (3)	
C15	0.2295 (3)	0.5639 (3)	0.63061 (15)	0.0539 (5)	
H15A	0.2683	0.5165	0.6940	0.081*	
H15B	0.0977	0.6081	0.6507	0.081*	
H15C	0.2795	0.6528	0.6075	0.081*	
C16	0.2474 (2)	0.69166 (19)	0.04489 (12)	0.0307 (3)	
C17	0.2142 (3)	0.7197 (2)	−0.06869 (14)	0.0419 (4)	
H17A	0.1961	0.8356	−0.0833	0.063*	
H17B	0.1068	0.6923	−0.0700	0.063*	
H17C	0.3186	0.6491	−0.1250	0.063*	

### Atomic displacement parameters (Å^2^)

**Table:**

	*U*^11^	*U*^22^	*U*^33^	*U*^12^	*U*^13^	*U*^23^
O1	0.0535 (8)	0.0781 (10)	0.0459 (8)	−0.0062 (7)	−0.0104 (6)	−0.0292 (7)
O2	0.0269 (6)	0.0580 (8)	0.0346 (6)	−0.0048 (5)	−0.0112 (5)	−0.0073 (5)
O3	0.0436 (6)	0.0307 (6)	0.0299 (6)	−0.0083 (5)	−0.0031 (5)	−0.0014 (4)
O4	0.0470 (7)	0.0484 (7)	0.0329 (6)	−0.0027 (5)	−0.0181 (5)	−0.0132 (5)
O5	0.0535 (7)	0.0332 (6)	0.0390 (6)	−0.0195 (5)	−0.0118 (5)	−0.0013 (5)
N1	0.0456 (8)	0.0279 (7)	0.0355 (7)	−0.0150 (6)	−0.0226 (6)	0.0018 (5)
C2	0.0348 (8)	0.0299 (7)	0.0302 (7)	−0.0130 (6)	−0.0154 (6)	0.0031 (6)
C3	0.0261 (7)	0.0362 (8)	0.0252 (7)	−0.0107 (6)	−0.0089 (5)	−0.0007 (6)
C4	0.0228 (7)	0.0319 (7)	0.0263 (7)	−0.0088 (6)	−0.0063 (5)	−0.0031 (6)
C5	0.0260 (7)	0.0290 (7)	0.0279 (7)	−0.0095 (6)	−0.0056 (6)	−0.0046 (6)
C6	0.0266 (7)	0.0313 (8)	0.0275 (7)	−0.0100 (6)	−0.0064 (6)	−0.0009 (6)
C7	0.0340 (8)	0.0353 (8)	0.0262 (7)	−0.0114 (6)	−0.0110 (6)	−0.0006 (6)
C8	0.0362 (8)	0.0306 (8)	0.0311 (8)	−0.0107 (6)	−0.0139 (6)	−0.0037 (6)
C9	0.0255 (7)	0.0296 (7)	0.0307 (7)	−0.0099 (6)	−0.0106 (6)	−0.0008 (6)
C10	0.0230 (7)	0.0307 (7)	0.0262 (7)	−0.0084 (6)	−0.0072 (5)	−0.0027 (6)
C11	0.0557 (11)	0.0401 (9)	0.0442 (9)	−0.0261 (8)	−0.0266 (8)	0.0126 (7)
C12	0.0389 (8)	0.0268 (7)	0.0292 (7)	−0.0073 (6)	−0.0129 (6)	0.0000 (6)
C13	0.0283 (9)	0.0824 (15)	0.0514 (11)	−0.0004 (9)	−0.0117 (8)	−0.0047 (10)
C14	0.0267 (7)	0.0353 (8)	0.0251 (7)	−0.0123 (6)	−0.0041 (5)	−0.0018 (6)
C15	0.0625 (12)	0.0587 (12)	0.0347 (9)	−0.0097 (10)	−0.0152 (8)	−0.0184 (8)
C16	0.0276 (7)	0.0323 (8)	0.0305 (8)	−0.0106 (6)	−0.0041 (6)	−0.0011 (6)
C17	0.0533 (10)	0.0393 (9)	0.0383 (9)	−0.0188 (8)	−0.0171 (8)	0.0095 (7)

### Geometric parameters (Å, º)

**Table:**

O1—C12	1.196 (2)	C7—H7	0.9300
O2—C12	1.3287 (19)	C8—C7	1.376 (2)
O2—C13	1.447 (2)	C8—H8	0.9300
O3—C14	1.2063 (19)	C9—C8	1.403 (2)
O4—C14	1.3401 (19)	C10—C4	1.474 (2)
O4—C15	1.4476 (19)	C10—C5	1.387 (2)
O5—C16	1.2211 (19)	C10—C9	1.422 (2)
N1—C2	1.4540 (19)	C11—H11A	0.9600
N1—C9	1.372 (2)	C11—H11B	0.9600
N1—H1	0.85 (2)	C11—H11C	0.9600
C2—C11	1.531 (2)	C13—H13A	0.9600
C2—C12	1.543 (2)	C13—H13B	0.9600
C3—C2	1.505 (2)	C13—H13C	0.9600
C3—C4	1.336 (2)	C15—H15A	0.9600
C3—H3	0.9300	C15—H15B	0.9600
C4—C14	1.496 (2)	C15—H15C	0.9600
C5—C6	1.402 (2)	C16—C17	1.509 (2)
C5—H5	0.9300	C17—H17A	0.9600
C6—C7	1.404 (2)	C17—H17B	0.9600
C6—C16	1.482 (2)	C17—H17C	0.9600
			
C12—O2—C13	116.35 (13)	C9—C10—C4	116.55 (13)
C14—O4—C15	116.25 (14)	C2—C11—H11A	109.5
C2—N1—H1	117.4 (13)	C2—C11—H11B	109.5
C9—N1—C2	120.53 (12)	C2—C11—H11C	109.5
C9—N1—H1	116.4 (13)	H11A—C11—H11B	109.5
N1—C2—C3	108.66 (12)	H11A—C11—H11C	109.5
N1—C2—C11	109.05 (12)	H11B—C11—H11C	109.5
N1—C2—C12	110.27 (12)	O1—C12—O2	124.00 (15)
C3—C2—C11	111.68 (13)	O1—C12—C2	124.45 (14)
C3—C2—C12	110.74 (11)	O2—C12—C2	111.53 (12)
C11—C2—C12	106.42 (13)	O2—C13—H13A	109.5
C2—C3—H3	119.0	O2—C13—H13B	109.5
C4—C3—C2	122.03 (13)	O2—C13—H13C	109.5
C4—C3—H3	119.0	H13A—C13—H13B	109.5
C3—C4—C10	120.21 (13)	H13A—C13—H13C	109.5
C3—C4—C14	119.01 (13)	H13B—C13—H13C	109.5
C10—C4—C14	120.62 (13)	O3—C14—O4	123.01 (14)
C6—C5—H5	119.0	O3—C14—C4	125.35 (14)
C10—C5—C6	121.90 (13)	O4—C14—C4	111.64 (13)
C10—C5—H5	119.0	O4—C15—H15A	109.5
C5—C6—C7	118.37 (13)	O4—C15—H15B	109.5
C5—C6—C16	118.55 (13)	O4—C15—H15C	109.5
C7—C6—C16	123.08 (13)	H15A—C15—H15B	109.5
C6—C7—H7	119.5	H15A—C15—H15C	109.5
C8—C7—C6	121.02 (13)	H15B—C15—H15C	109.5
C8—C7—H7	119.5	O5—C16—C6	120.92 (14)
C7—C8—C9	120.37 (13)	O5—C16—C17	119.68 (14)
C7—C8—H8	119.8	C6—C16—C17	119.41 (13)
C9—C8—H8	119.8	C16—C17—H17A	109.5
N1—C9—C8	120.72 (13)	C16—C17—H17B	109.5
N1—C9—C10	119.52 (13)	H17A—C17—H17B	109.5
C8—C9—C10	119.69 (13)	C16—C17—H17C	109.5
C5—C10—C4	124.87 (13)	H17A—C17—H17C	109.5
C5—C10—C9	118.57 (13)	H17B—C17—H17C	109.5
			
C13—O2—C12—O1	−2.3 (3)	C10—C4—C14—O4	−159.98 (12)
C13—O2—C12—C2	176.23 (15)	C10—C5—C6—C7	1.0 (2)
C15—O4—C14—O3	3.7 (2)	C10—C5—C6—C16	−179.06 (13)
C15—O4—C14—C4	−176.03 (14)	C5—C6—C7—C8	−2.2 (2)
C9—N1—C2—C3	41.36 (19)	C16—C6—C7—C8	177.88 (14)
C9—N1—C2—C11	163.31 (14)	C5—C6—C16—O5	6.5 (2)
C9—N1—C2—C12	−80.19 (17)	C5—C6—C16—C17	−173.14 (13)
C2—N1—C9—C8	154.73 (14)	C7—C6—C16—O5	−173.48 (14)
C2—N1—C9—C10	−28.4 (2)	C7—C6—C16—C17	6.8 (2)
N1—C2—C12—O1	−33.1 (2)	C9—C8—C7—C6	0.7 (2)
N1—C2—C12—O2	148.35 (13)	N1—C9—C8—C7	178.80 (14)
C3—C2—C12—O1	−153.45 (17)	C10—C9—C8—C7	2.0 (2)
C3—C2—C12—O2	28.05 (17)	C5—C10—C4—C3	−167.68 (14)
C11—C2—C12—O1	85.0 (2)	C5—C10—C4—C14	17.1 (2)
C11—C2—C12—O2	−93.51 (15)	C9—C10—C4—C3	13.5 (2)
C4—C3—C2—N1	−28.33 (19)	C9—C10—C4—C14	−161.74 (13)
C4—C3—C2—C11	−148.66 (14)	C4—C10—C5—C6	−177.15 (13)
C4—C3—C2—C12	92.93 (16)	C9—C10—C5—C6	1.6 (2)
C2—C3—C4—C10	2.8 (2)	C4—C10—C9—N1	−1.1 (2)
C2—C3—C4—C14	178.09 (12)	C4—C10—C9—C8	175.78 (12)
C3—C4—C14—O3	−155.01 (15)	C5—C10—C9—N1	−179.95 (13)
C3—C4—C14—O4	24.69 (19)	C5—C10—C9—C8	−3.1 (2)
C10—C4—C14—O3	20.3 (2)		

### Hydrogen-bond geometry (Å, º)

**Table:**

*D*—H···*A*	*D*—H	H···*A*	*D*···*A*	*D*—H···*A*
N1—H1···O5^i^	0.85 (2)	2.10 (2)	2.9223 (19)	166 (2)

Symmetry code: (i) *x*, *y*−1, *z*.
